# Interpretable machine learning for predicting 28-day all-cause in-hospital mortality for hypertensive ischemic or hemorrhagic stroke patients in the ICU: a multi-center retrospective cohort study with internal and external cross-validation

**DOI:** 10.3389/fneur.2023.1185447

**Published:** 2023-08-08

**Authors:** Jian Huang, Huaqiao Chen, Jiewen Deng, Xiaozhu Liu, Tingting Shu, Chengliang Yin, Minjie Duan, Li Fu, Kai Wang, Song Zeng

**Affiliations:** ^1^Emergency Department, The Second Affiliated Hospital of Chongqing Medical University, Chongqing, China; ^2^The Graduate School of Guangxi University of Traditional Chinese Medicine, Nanning, China; ^3^Department of Cardiology, The First Affiliated Hospital of Chongqing Medical University, Chongqing, China; ^4^Department of Neurosurgery, Xiu Shan People's Hospital, Chongqing, China; ^5^Department of Critical Care Medicine, Beijing Shijitan Hospital, Capital Medical University, Beijing, China; ^6^Department of Cardiology, Daping Hospital, The Third Military Medical University (Army Medical University), Chongqing, China; ^7^Faculty of Medicine, Macau University of Science and Technology, Taipa, Macao SAR, China; ^8^College of Medical Informatics, Chongqing Medical University, Chongqing, China; ^9^Key Laboratory of Novel Materials for Sensor of Zhejiang Province, College of Materials and Environmental Engineering, Hangzhou Dianzi University, Hangzhou, China; ^10^Department of Neurology, The Second Affiliated Hospital of Xuzhou Medical University, Xuzhou, Jiangsu, China

**Keywords:** machine learning, hypertensive, stroke, all-cause mortality, interpretable prediction model, SHAP

## Abstract

**Background:**

Timely and accurate outcome prediction plays a critical role in guiding clinical decisions for hypertensive ischemic or hemorrhagic stroke patients admitted to the ICU. However, interpreting and translating the predictive models into clinical applications are as important as the prediction itself. This study aimed to develop an interpretable machine learning (IML) model that accurately predicts 28-day all-cause mortality in hypertensive ischemic or hemorrhagic stroke patients.

**Methods:**

A total of 4,274 hypertensive ischemic or hemorrhagic stroke patients admitted to the ICU in the USA from multicenter cohorts were included in this study to develop and validate the IML model. Five machine learning (ML) models were developed, including artificial neural network (ANN), gradient boosting machine (GBM), eXtreme Gradient Boosting (XGBoost), logistic regression (LR), and support vector machine (SVM), to predict mortality using the MIMIC-IV and eICU-CRD database in the USA. Feature selection was performed using the Least Absolute Shrinkage and Selection Operator (LASSO) algorithm. Model performance was evaluated based on the area under the curve (AUC), accuracy, positive predictive value (PPV), and negative predictive value (NPV). The ML model with the best predictive performance was selected for interpretability analysis. Finally, the SHapley Additive exPlanations (SHAP) method was employed to evaluate the risk of all-cause in-hospital mortality among hypertensive ischemic or hemorrhagic stroke patients admitted to the ICU.

**Results:**

The XGBoost model demonstrated the best predictive performance, with the AUC values of 0.822, 0.739, and 0.700 in the training, test, and external cohorts, respectively. The analysis of feature importance revealed that age, ethnicity, white blood cell (WBC), hyperlipidemia, mean corpuscular volume (MCV), glucose, pulse oximeter oxygen saturation (SpO_2_), serum calcium, red blood cell distribution width (RDW), blood urea nitrogen (BUN), and bicarbonate were the 11 most important features. The SHAP plots were employed to interpret the XGBoost model.

**Conclusions:**

The XGBoost model accurately predicted 28-day all-cause in-hospital mortality among hypertensive ischemic or hemorrhagic stroke patients admitted to the ICU. The SHAP method can provide explicit explanations of personalized risk prediction, which can aid physicians in understanding the model.

## 1. Introduction

Stroke is the second most common cause of death and the third leading cause of disability worldwide, imposing a substantial economic burden in terms of healthcare costs and reduced productivity (1, 2). Low- and lower-middle-income countries bear the majority of the global stroke burden, accounting for 86% of stroke-related fatalities (2). The incidence of cerebral ischemic stroke is significantly higher than that of hemorrhagic stroke, with ischemic stroke being the more prevalent type. Ischemic stroke accounts for ~87% of all stroke cases, while intracerebral hemorrhage and subarachnoid hemorrhage contribute to 10 and 3% of strokes, respectively (3). Hypertension is the most prevalent modifiable risk factor for stroke in both industrialized and developing nations (4). It is an indicator of poor prognosis in 70% or more of individuals with acute ischemic or hemorrhagic stroke (5). Moreover, the intricate interaction between hypertension and other modifiable risk factors, including smoking, high body mass index, diabetes mellitus, and high cholesterol, substantially increases the overall risk of cardiovascular and cerebrovascular diseases in individuals (4).

The prevalence of patients with hypertension among stroke patients is high, and there is no prediction model for predicting 28-day in-hospital mortality for hypertensive ischemic or hemorrhagic stroke patients in the ICU. Precise and adaptable assessment tools play a crucial role in the early identification of high-risk patients in the ICU. Conventional approaches, such as the Cox proportional hazard model, are inefficient in examining the intricate non-linear relationships within the data (6, 7). Machine learning (ML) is increasingly utilized in medicine to quantify risk, identify predictors, and develop highly accurate prediction models for diagnosis and prognosis (8, 9).

In the present study, five ML models, including artificial neural network (ANN), gradient boosting machine (GBM), eXtreme Gradient Boosting (XGBoost), logistic regression model (LR), and support vector machine (SVM), were constructed to explore the risk factors of hypertensive ischemic or hemorrhagic stroke patients in the ICU and to support clinical decision-making based on clinical characteristics. Additionally, an interpretable machine learning (IML) approach was employed to predict the 28-day in-hospital mortality of hypertensive patients with ischemic or hemorrhagic stroke who were admitted to the ICU, using SHapley Additive exPlanations (SHAP) values and feature significance.

## 2. Materials and methods

### 2.1. Data source

Data for this study were collected from the Medical Information Mart for Intensive Care IV (MIMIC-IV) database (https://mimic.physionet.org/, certification ID: 42039823) and the Collaborative Research Database (eICU-CRD, https://eicu-crd.mit.edu/). The MIMIC-IV database is a publicly accessible intensive care database that comprises de-identified clinical data from over 70,000 ICU hospitalizations in the USA from 2008 to 2019 (10). The eICU-CRD database is a multi-center intensive care database that is made available to the public by Philips Healthcare in collaboration with the MIT Laboratory for Computational Physiology. It contains de-identified clinical data for over 200,000 patients who were admitted to the ICU from 2014 to 2015 (11). The de-identified health information of patients was collected, and informed consent was not required for this study.

### 2.2. Study population and outcome

**Figure 1:**
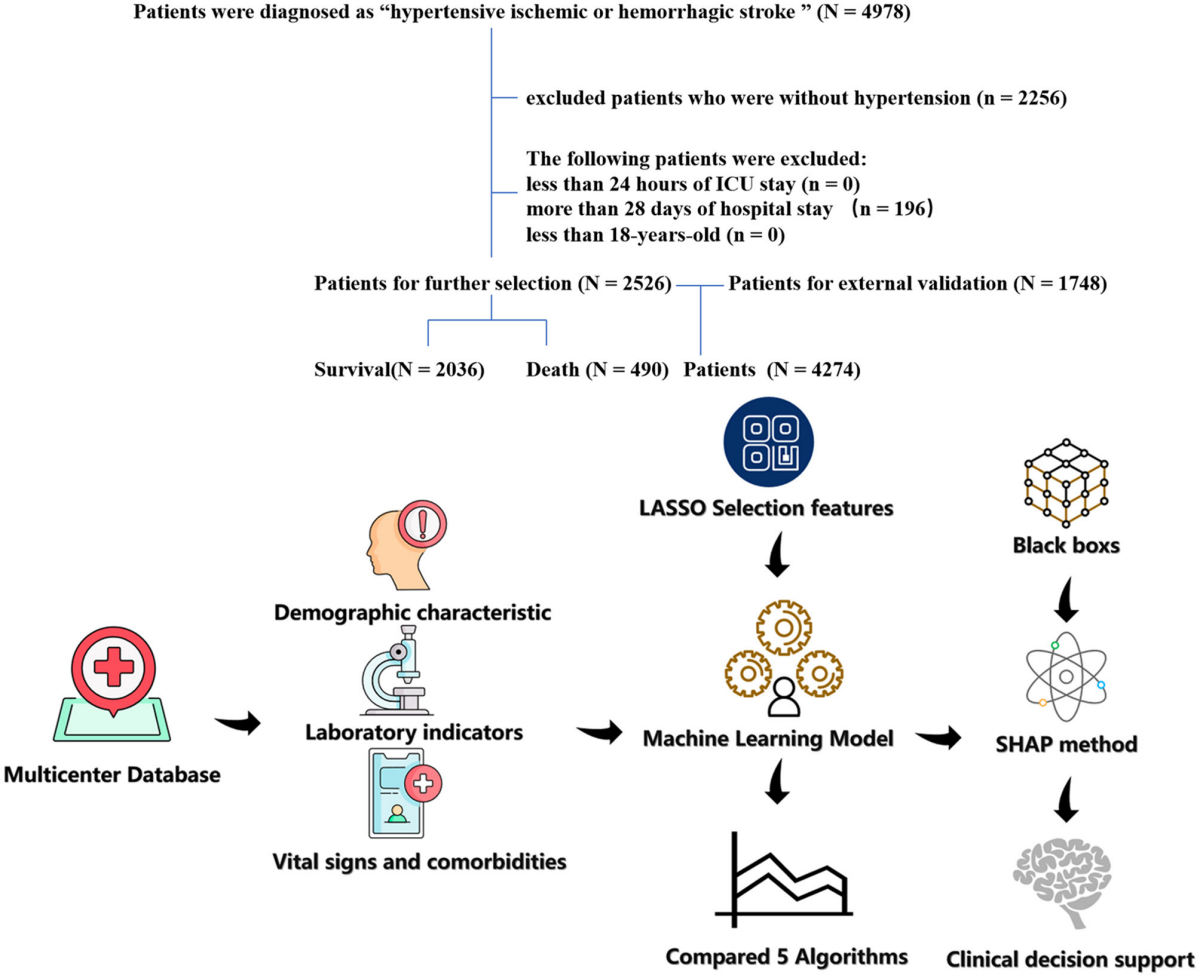
The flow chart of patient selection. The study included 2,526 patients with hypertension and hypertension ischemic stroke.

Hypertensive ischemic or hemorrhagic stroke patients in the last ICU stay were enrolled in the study cohort. Ischemic or hemorrhagic stroke and hypertensive patients were found to use diagnosis codes from the International Classification of Diseases, Ninth Revision (ICD-9). The screening process of the patients included in this study is shown in Figure 1. The exclusion criteria of this study include the following: (1) <24 h of ICU stay; (2) more than 28 days of ICU stay; (3) patients <18 years of age; and (4) patients with missing data (death). The primary outcome of this study is 28-day in-hospital mortality in the ICU.

### 2.3. Data extraction and preprocessing

Clinical data of all participants were collected from the eICU-CRD and MIMIC-IV database based on previously published literature with relevant topics (12, 13). A total of 41 predictor features consisting of demographics, laboratory tests, and co-morbidities were analyzed. Features with missing values of more than 30% were excluded to guarantee a higher accuracy of the outcome, and the k-Nearest Neighbors (kNN) imputation was applied to impute the missing values. The R package “DMwR2” was used for kNN imputation.

### 2.4. Construction of the machine learning model

In our study, models were developed to predict the 28-day in-hospital mortality of hypertensive ischemic or hemorrhagic stroke patients using five widely used algorithms, including ANN, GBM, LR, XGBoost, and SVM. All continuous variables were rescaled to have a distribution with a mean of 0 and a standard deviation of 1 using scale transformation to increase the stability of the prediction models. To choose the optimal prediction model for each algorithm with various tuning parameters, 5-fold cross-validation was applied to the ML models that needed tuning. The accuracy or receiver operating characteristic (ROC) was chosen as the metric during the search procedure. The testing set was solely utilized for model evaluation after concluding the complete model selection and training procedure. It was not employed during model tuning.

### 2.5. Model assessment

The confusion matrix metrics of accuracy and area under the receiver operating characteristic curve (AUROC) were used to assess the final models. Based on the prediction probabilities, the ROC curves were developed. Then, the model with the best predictive performance was identified by comparing the AUC values of the models in the testing data set.

### 2.6. Interpretation analysis

#### 2.6.1. Feature importance

Feature ranking evaluation refers to a method of measuring the significance of each feature in the feature set based on its impact on the final classification outcome. Feature importance was measured using the “shapviz” package, which describes any classifier's predictions understandably and faithfully by learning an understandable model locally around the prediction. Relative variable importance was computed and presented to seek out the effect of features on the predictive models.

#### 2.6.2. Shapley additive explanation (SHAP) value

The SHAP value of features was evaluated using the “shapviz” package. We selected SHAP summary, SHAP force plot, and SHAP waterfall to evaluate the SHAP value of features, which would increase the clinical utility of the predictive models.

### 2.7. Statistical analysis

The original dataset was randomly divided into the training set (*n* = 2,031) for developing the models and the testing set (*n* = 495) for evaluating the models' performance, based on a ratio of 8:2 in the eICU-CRD database. External validation was performed using the MIMIC-IV database. In the training set, testing set, and MIMIC-IV database, continuous data with normal distribution were demonstrated as the mean with standard errors, continuous data with non-normal distribution were demonstrated as the median with interquartile range (IQR), and categorical data were demonstrated as the frequency (percentage). A chi-squared test was performed to compare the qualitative features. To regularize the results of the statistical analysis for potential confounding factors, LASSO regression analysis was performed to predict 28-day in-hospital mortality in hypertensive patients with ischemic and hemorrhagic stroke. This enhances the prediction accuracy and the interpretation ability of a statistical model and is appropriate for high-dimensional data reduction. To guarantee minimized autocorrelation, features having non-zero coefficients were chosen for the additional analysis in the LASSO regression model. R 4.1.3 and Rstudio 1.1.463 were used for all statistical analyses. The R package “caret” was used to pre-process the data, tune the parameters, and train the model. The R package “shapviz” was used to evaluate the SHAP value and feature importance. The R package “rcs” was used to evaluate the cutoff value of features. A forest plot was performed using the package “forestplot.” The LASSO and logistic regression analyses were performed using the R package “glmnet.” To evaluate the effectiveness of each model, the ROC curve analysis and the AUC were computed using the “pROC” and ggplot2 packages. All *P*-values were two-sided, and features with a *P*-value of <0.05 were deemed statistically significant.

## 3. Results

### 3.1. Patient characteristics

This study comprised 2,526 hypertensive ischemic or hemorrhagic stroke patients who were admitted to the ICU. Patient characteristics are shown in Table 1. In total, the median age was 71 years, and 1,256 patients (50%) were women. Furthermore, 269 (11%) patients had myocardial ischemia (MI), 255 (10%) had chronic heart failure (CHF), 128 (5%) had dementia, 409 (16%) had chronic obstructive pulmonary disease (COPD), 599 (24%) had diabetes, 738 (29%) had atrial fibrillation (AF), and 1,330 (51%) had hyperlipidemia. The baseline characteristics of the training and testing sets did not differ significantly. In this study, 490 (19%) patients experienced in-hospital death. Baseline data for external validation sets are shown in Supplementary Table 1.

**Table 1:** Baseline characteristics, laboratory parameters, vital signs, and statistical results of patients with hypertension and ischemic stroke in the training and testing sets.

**Variables**	**Total (*n* = 2,526)**	**Train set (*n* = 2,031)**	**Test set (*n* = 495)**	***P***
Hemorrhagic stroke, *n* (%)				0.75
No	1,269 (50)	1,024 (50)	245 (49)	
Yes	1,257 (50)	1,007 (50)	250 (51)	
Gender, *n* (%)				0.812
Female	1,256 (50)	1,007 (50)	249 (50)	
Male	1,270 (50)	1,024 (50)	246 (50)	
Ethnicity, *n* (%)				0.229
Non-white	955 (38)	780 (38)	175 (35)	
White	1,571 (62)	1,251 (62)	320 (65)	
Age, years	71.2 (60.91, 81.6)	71.27 (61.26, 81.67)	70.55 (58.76, 81.31)	0.32
HR, per minute	80 (70, 92)	80 (70, 92)	80 (70, 93.5)	0.473
SBP, mmHg	140 (125, 155)	140 (125, 155)	138 (123, 154)	0.302
DBP, mmHg	74.5 (63, 86)	74 (63, 86)	75 (64, 87)	0.493
DBP, mmHg	92.5 (81, 104)	93 (81, 103)	92 (83, 104.5)	0.686
RR, per minute	18 (15, 21)	18 (15, 21)	18 (15, 22)	0.774
Temperature, °C	36.78 (36.5, 37.06)	36.78 (36.44, 37.06)	36.78 (36.5, 37.06)	0.372
SPO_2_, %	98 (96, 100)	98 (96, 100)	98 (96, 100)	0.798
INR	1.1 (1, 1.2)	1.1 (1, 1.2)	1.1 (1, 1.3)	0.139
PT, second	12.3 (11.4, 13.8)	12.3 (11.4, 13.7)	12.3 (11.4, 14.25)	0.274
APTT, second	27.9 (25.3, 31.5)	27.9 (25.4, 31.5)	28 (25.2, 31.3)	0.823
WBC, ^*^10^9^/*L*	10.2 (7.8, 13.5)	10.2 (7.9, 13.4)	10.2 (7.55, 13.6)	0.729
RBC, 10^9^/*L*	4.25 (3.8, 4.65)	4.26 (3.8, 4.65)	4.23 (3.79, 4.66)	0.862
Hemoglobin, g/L	128 (114, 140)	128 (114, 141)	127 (112, 140)	0.448
Hematocrit, %	38.6 (34.6, 42)	38.6 (34.6, 42)	38.8 (34.35, 41.95)	0.68
MCH, pg	30.3 (28.9, 31.6)	30.3 (29, 31.6)	30.2 (28.9, 31.5)	0.358
MCHC, g/L	332 (323, 342)	333 (323, 342)	332 (322, 342)	0.43
MCV, fl	91 (87, 94)	91 (87, 94)	90 (87, 94)	0.306
RDW, %	13.6 (13, 14.6)	13.6 (13, 14.5)	13.6 (13, 14.7)	0.451
Platelets, ^*^10^1^1/*L*	215 (169, 268)	215 (170, 266)	217 (166, 274)	0.535
Anion gap	15 (13, 17)	15 (13, 17)	15 (13, 17)	0.34
Creatinine, mg/dL	0.9 (0.7, 1.1)	0.9 (0.7, 1.1)	0.9 (0.7, 1.1)	0.81
BUN, mg/dL	17 (13, 22)	17 (13, 22)	16 (13, 22)	0.377
Calcium, mg/dL	8.8 (8.4, 9.2)	8.8 (8.4, 9.2)	8.85 (8.4, 9.2)	0.525
Potassium, mmol/L	4 (3.7, 4.4)	4 (3.7, 4.4)	4 (3.7, 4.4)	0.288
Sodium, mmol/L	139 (137, 141)	139 (137, 141)	139 (137, 141)	0.816
Chloride, mmol/L	103 (100, 106)	103 (100, 106)	103 (100, 106)	0.713
Bicarbonate, mmol/L	24 (21, 26)	24 (21, 26)	24 (22, 26)	0.246
Glucose, mg/dL	130 (108, 163)	130 (108, 162)	133 (105, 169)	0.517
MI, *n* (%)				0.772
No	2,257 (89)	1,817 (89)	440 (89)	
Yes	269 (11)	214 (11)	55 (11)	
CHF, *n* (%)				0.564
No	2,271 (90)	1,822 (90)	449 (91)	
Yes	255 (10)	209 (10)	46 (9)	
Dementia, *n* (%)				0.581
No	2,398 (95)	1,931 (95)	467 (94)	
Yes	128 (5)	100 (5)	28 (6)	
COPD, *n* (%)				0.366
No	2,117 (84)	1,695 (83)	422 (85)	
Yes	409 (16)	336 (17)	73 (15)	
Diabetes, *n* (%)				0.057
No	1,927 (76)	1,566 (77)	361 (73)	
Yes	599 (24)	465 (23)	134 (27)	
Stroke, *n* (%)				0.68
No	1,718 (68)	1,377 (68)	341 (69)	
Yes	808 (32)	654 (32)	154 (31)	
AF, *n* (%)				0.276
No	1,788 (71)	1,448 (71)	340 (69)	
Yes	738 (29)	583 (29)	155 (31)	
Hyperlipidemia, *n* (%)				0.707
No	1,226 (49)	990 (49)	236 (48)	
Yes	1,300 (51)	1,041 (51)	259 (52)	
APSIII	37 (27, 50)	37 (27, 50.5)	36 (27, 49)	0.457
GCS (minimum)	12 (8, 14)	12 (8, 14)	13 (9, 14)	0.196
Death, *n* (%)				0.749
No	2,036 (81)			
Yes	490 (19)			

All continuous variables are presented as median (Q1, Q3).

HR, heart rate; SBP, systolic blood pressure; DBP, diastolic blood pressure; RR, respiratory rate; SpO_2_, peripheral oxygen saturation; INR, international normalized ratio; PT, prothrombin time; APTT, activated partial thromboplastin time; WBC, white blood cell; RBC, red blood cell; MCH, mean corpuscular hemoglobin; MCHC, mean corpuscular hemoglobin concentration; MCV, mean corpuscular volume; RDW, red cell distribution width; BUN, blood urea nitrogen; MI, myocardial infarction; CHF, congestive heart failure; COPD, chronic obstructive pulmonary disease; AF, atrial fibrillation; APSIII, Acute Physiology Score III; GCS, Glasgow Coma Score.

### 3.2. Selection of predictors

**Figure 2:**
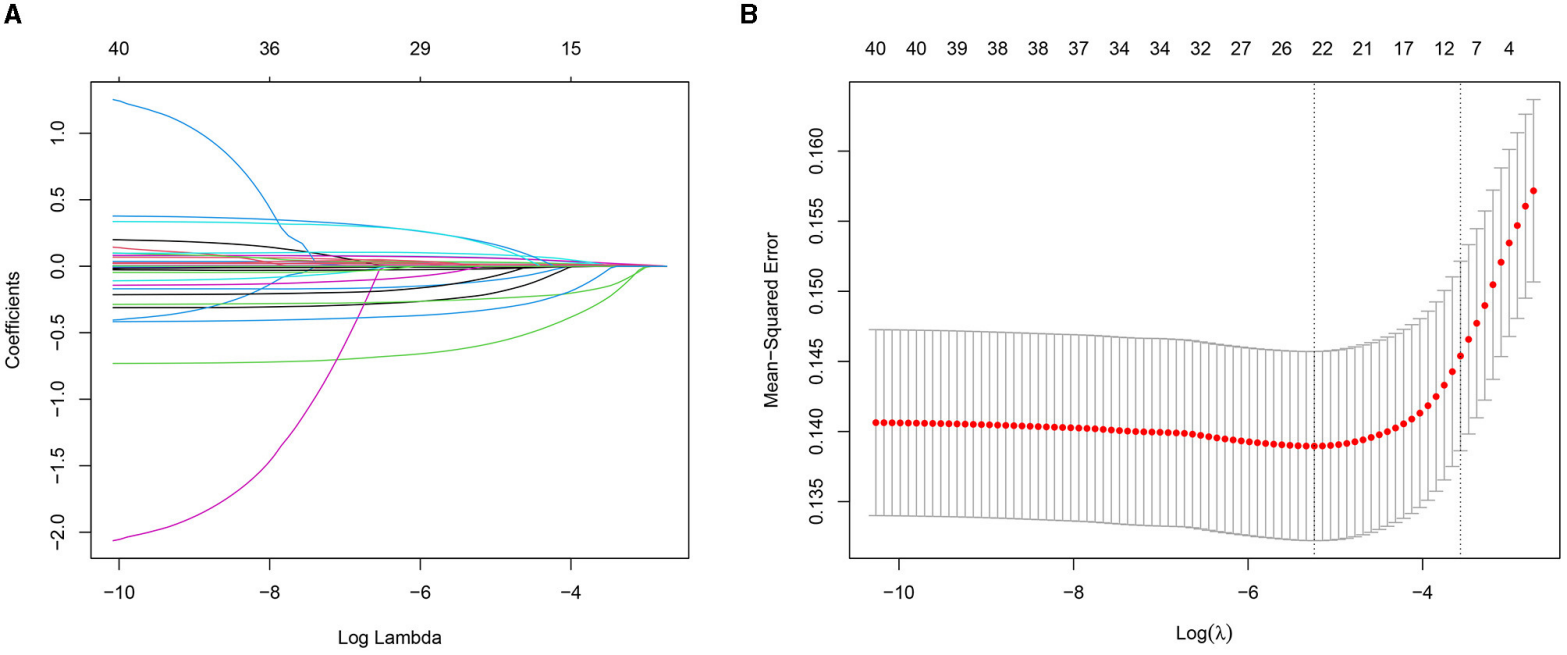
The result of the Least Absolute Shrinkage and Selection Operator (LASSO) method for filtering variables. **(A)** Coefficients of all predictors gradually returning to zeros by used 10-fold cross-validation. **(B)** 11 predictors with non-zero coefficients at the rightmost dashed line.

In the LASSO method, the penalty on the β-coefficients was controlled by the tuning parameter λ (λ = 0.02574252; lambda.1se; Figure 2). Eleven features with non-zero coefficients were selected, including ethnicity, age, peripheral oxygen saturation (SpO_2_), white blood cell (WBC), mean corpuscular volume (MCV), red blood cell distribution width (RDW), bicarbonate, blood urea nitrogen (BUN), calcium, glucose, and hyperlipidemia.

### 3.3. Model performance

**Figure 3:**
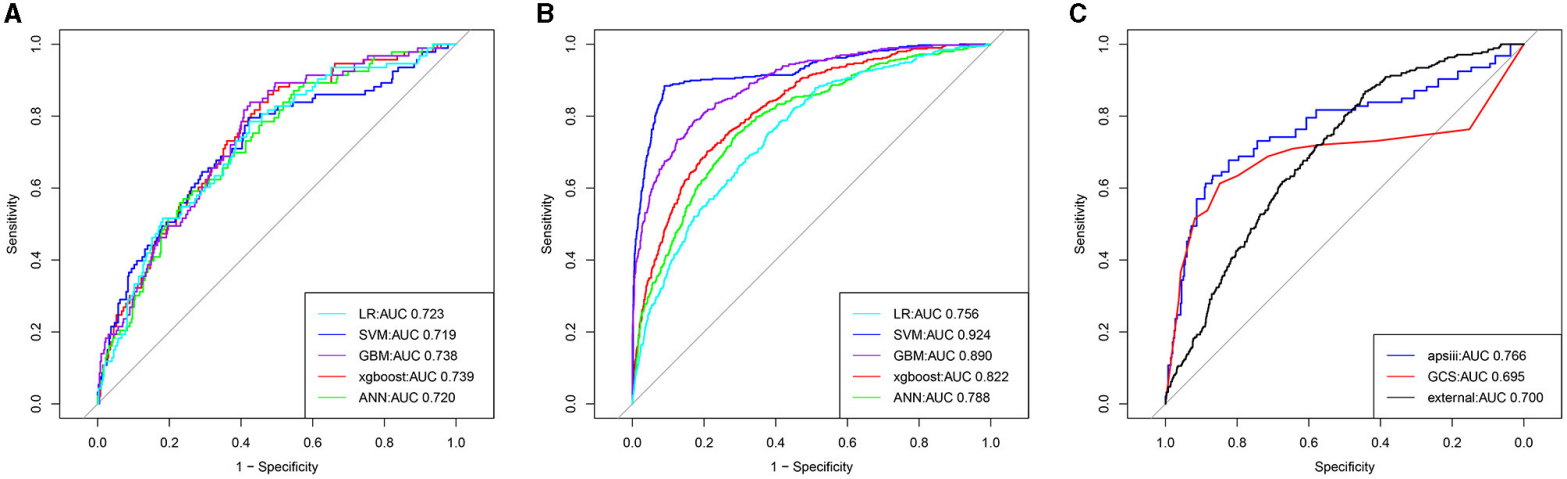
The receiver operator characteristic (ROC) curves for the ML models predict all-cause mortality in patients with hypertension and ischemic stroke (the training, testing, and external validation sets). The ROC curves of the nine ML models predicting all-cause mortality in the testing **(A)**, training **(B)**, and external validation sets **(C)**, respectively.

**Figure 4:**
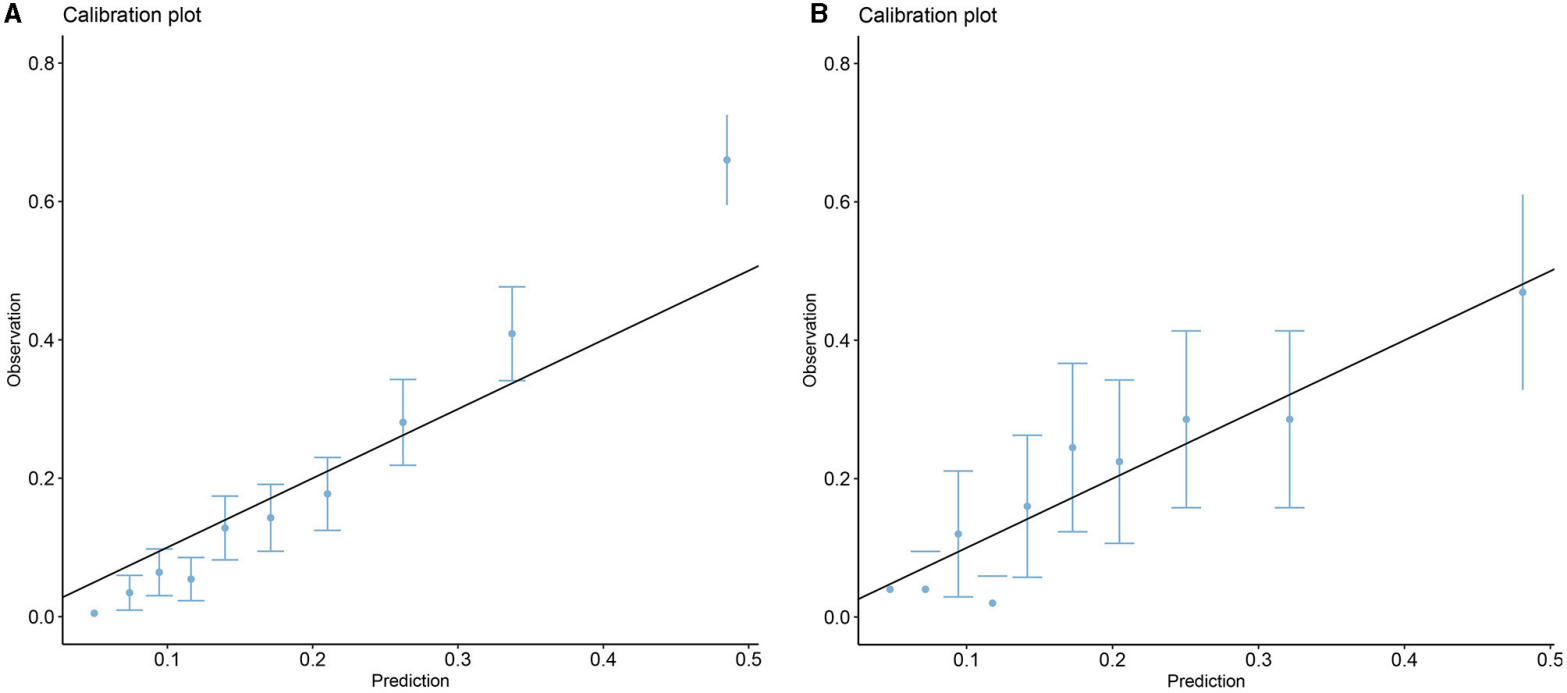
Calibration plots of the XGBoost model in the training **(A)** and testing sets **(B)**.

Five models including ANN, GBM, LR, XGBoost, and SVM were developed. Each model was evaluated by the AUROC and accuracy. All models had accuracy values of 0.80 and above in the testing set. The accuracy of the ANN, GBM, LR, XGBoost, and SVM in the testing (training) set was 0.802 (0.833), 0.808 (0.874), 0.804 (0.814), 0.818 (0.822), and 0.816 (0.871), respectively (Table 2). The AUROCs of the ANN, GBM, LR, XGBoost, and SVM in the testing set were 0.720, 0.738, 0.723, 0.739, and 0.719, respectively. The ROC curves and AUROC of different models in the testing and training sets are shown in Figures 3A, B. Meanwhile, the AUROCs of the Acute Physiology Score III (APS III) and Glasgow Coma Scale (GCS) scoring were 0.766 and 0.695 in the testing set, and the AUROC of the XGBoost model was 0.700 in the MIMIC-IV database (Figure 3C). Figure 4 shows that the prediction of the XGBoost model in the training and testing sets is in good agreement with the actual outcome, and the model calibration performance is good.

**Table 2:** Assessment of each model by accuracy (95%CI), PPV, and NPV in the training and testing sets.

	**Train set**					**Test set**				
	**ANN**	**GBM**	**LR**	**Xgboost**	**SVM**	**ANN**	**GBM**	**LR**	**Xgboost**	**SVM**
Accuracy	0.833	0.874	0.814	0.822	0.871	0.802	0.808	0.804	0.818	0.816
Lower 95% CI	0.816	0.858	0.797	0.804	0.856	0.764	0.771	0.766	0.781	0.779
Upper 95% CI	0.849	0.888	0.831	0.838	0.885	0.836	0.842	0.838	0.851	0.849
PPV	0.713	0.846	0.589	0.754	0.947	0.439	0.474	0.428	0.600	0.600
NPV	0.842	0.877	0.828	0.824	0.865	0.835	0.836	0.826	0.825	0.821

ANN, artificial neural network; GBM, gradient boosting machine; LR, logistic regression; xgboost, eXtremeGradient Boosting; SVM, support vector machine; CI, confidence interval; PPV, positive predict value; NPV, negative predict value.

### 3.4. Feature importance

**Figure 5:**
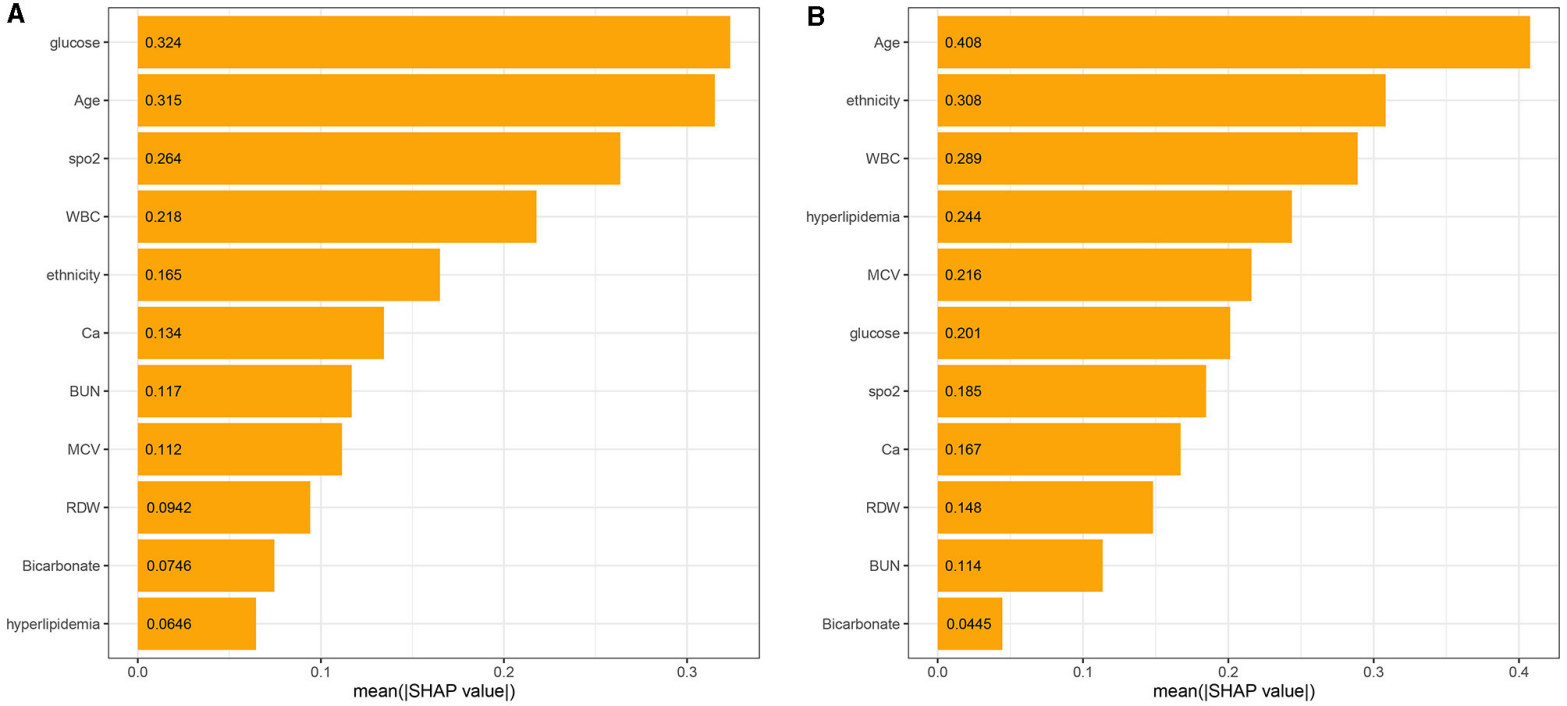
The model's interpretation: **(A)** the importance ranking of the top 11 variables according to the mean (SHAP value) in the XGBoost model and **(B)** the importance ranking of the top 11 risk factors in the LR model. The higher the SHAP value of a feature, the higher the risk of death for the patient.

The 11 most important features of the best ML model were calculated, as shown in Figure 5A. The characteristics of the laboratory test, including glucose, WBC, calcium, BUN, MCV, RDW, and bicarbonate, vital signs, such as SpO_2_, and comorbidity, such as hyperlipidemia, significantly affected most predictive models. Demographic characteristics including age and ethnicity also significantly affected most predictive models.

Meanwhile, the 11 most important features of the LR model in the ascending order were age, ethnicity, WBC, hyperlipidemia, MCV, glucose, SpO_2_, calcium, RDW, BUN, and bicarbonate, as shown in Figure 5B. There were similarities in the most important features between the XGBoost and the LR model (Figure 5).

### 3.5. SHAP values of features

**Figure 6:**
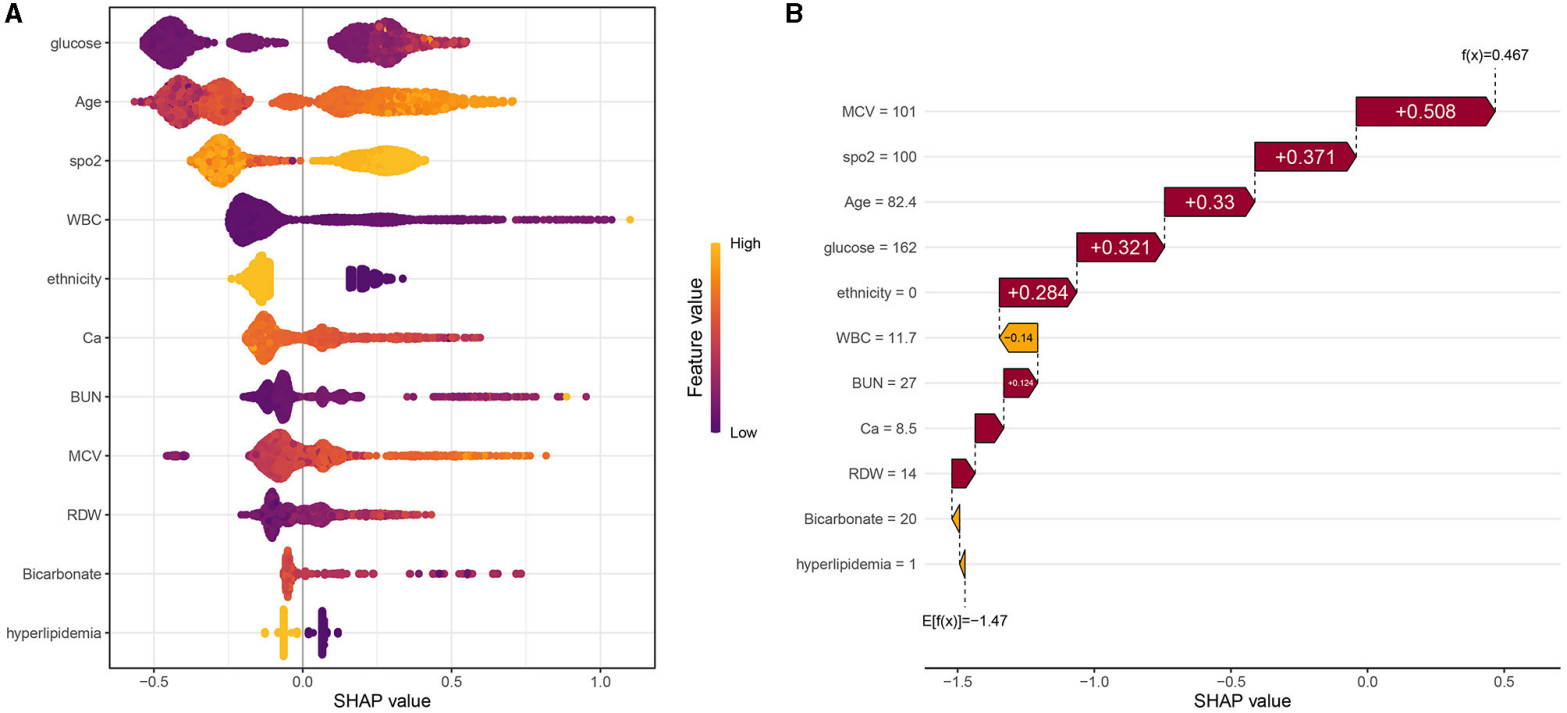
**(A)** Scatter plot of feature values and SHAP values. **(B)** Consent waterfall plot showing an example of interpretability analysis for a patient. The yellow part of the feature value represents a higher value. The purple part of the feature value represents a lower value.

**Figure 7:**
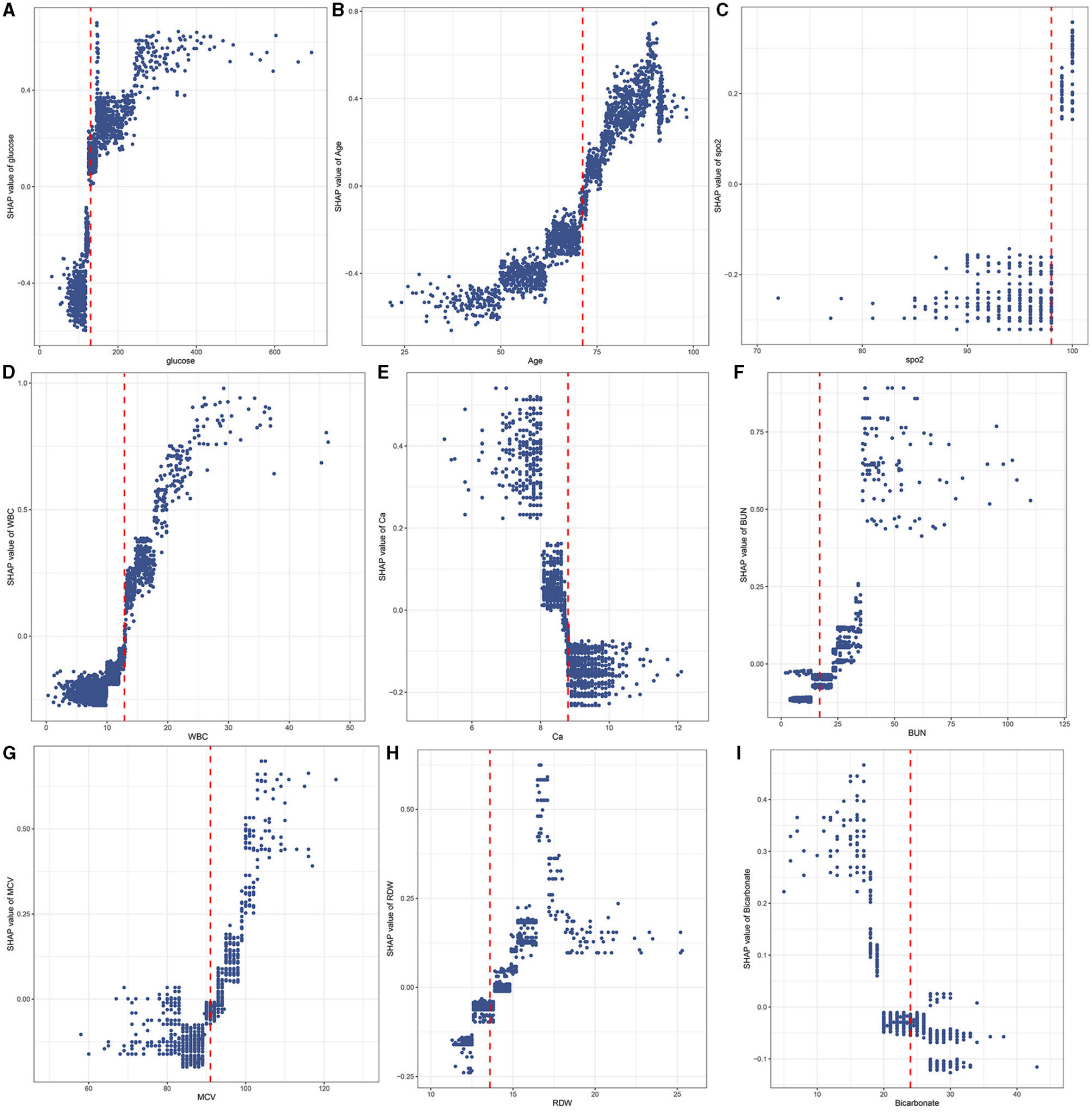
A scatter plot of variable values vs. SHAP values. Glucose **(A)**; Age **(B)**; SpO_2_ **(C)**; WBC **(D)**; Ca **(E)**; BUN **(F)**; MCV **(G)**; RDW **(H)**; Bicarbonate **(I)**.

The SHAP values of features are summarized in Figure 6A. The SHAP values of patients' features, including RDW of 14%, calcium of 8.5 mg/dL, BUN of 27 mg/dL, ethnicity of 0, glucose of 162 mg/dL, age of 82.4 years, SpO_2_ of 100%, MCV of 101 fl, WBC of 11.7^*^10^9^/*L*, bicarbonate of 20 mmol/L, and hyperlipidemia of 1, are shown in Figure 6B. Furthermore, the features based on their contribution to the model are glucose, age, SpO_2_, WBC, ethnicity, calcium, BUN, MCV, RDW, bicarbonate, and hyperlipidemia, in descending order. Figure 7 shows that glucose, age, WBC, BUN, MCV, and RDW are positively correlated with SHAP values, suggesting that the higher their levels, the greater the SHAP values, which is conducive to the occurrence of outcome events.

### 3.6. Cutoff values of features

**Figure 8:**
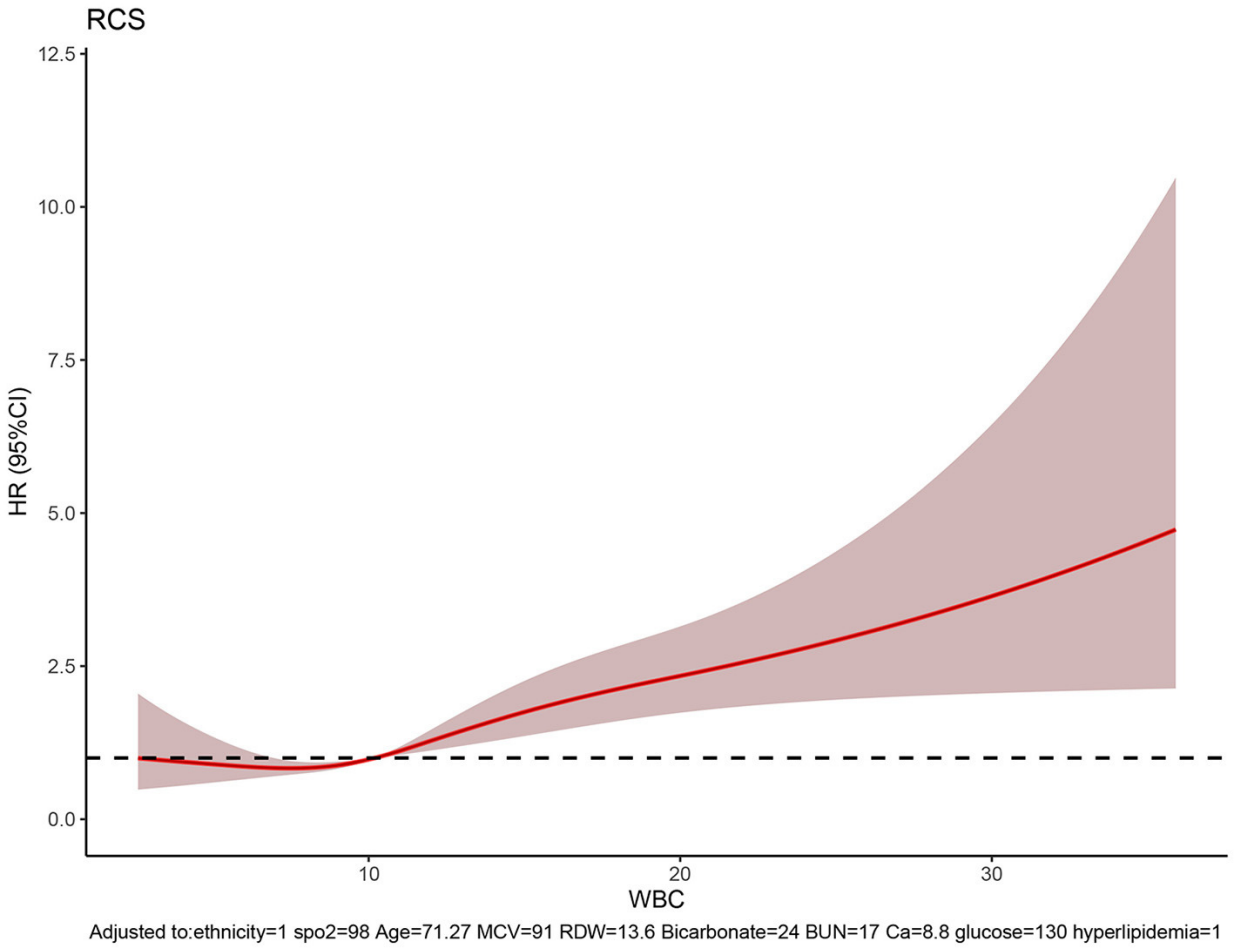
RCS showing the cutoff values of features, including SpO_2_ of 98%, age of 71.27 years, MCV of 91 fl, RDW of 13.6%, bicarbonate of 24 mmol/L, BUN of 17 mg/dL, calcium of 8.8 mg/dL, and glucose of 130 mg/dL.

The RCS showed the cutoff values of features, including SpO_2_ of 98%, age of 71.27 years, MCV of 91 fl, RDW of 13.6%, bicarbonate of 24 mmol/L, BUN of 17 mg/dL, calcium of 8.8 mg/dL, and glucose of 130 mg/dL after adjustment for covariates (Figure 8). These cutoff values were consistent with the tendency of SHAP values (Figure 7). The calibration plot showed a high degree of predictability between the actual and predicted probabilities (Figure 4). Univariate adjusted RCS analysis of the relationship between SpO_2_ and the outcome showed that the cutoff value was 92.6%, which indicated a low level of oxygen saturation.

### 3.7. Subgroup analysis of ischemic stroke and hemorrhagic stroke

Important features such as ethnicity, SpO_2_, age, MCV, RDW, BUN, calcium, glucose, hyperlipidemia, and WBC were independent risk factors in the ischemic stroke subgroup. Furthermore, important features such as ethnicity, age, MCV, RDW, calcium, hyperlipidemia, and WBC were independent risk factors in the intracerebral hemorrhage group (Table 3).

**Table 3:** A subgroup analysis of ischemic stroke and hemorrhagic stroke.

	**Ischemic stroke**		**Hemorrhagic stroke**	
	**OR (95% CI)**	***P***	**OR (95% CI)**	***P***
Age	1.04 (1.03–1.06)	0	1.03 (1.01–1.04)	0
Ethnicity	0.51 (0.36–0.72)	0	0.51 (0.36–0.73)	0
SpO_2_	1.14 (1.06–1.22)	0	1.14 (0.98–1.10)	0.227
Glucose	1.01 (1.00–1.01)	0	1.00 (1.00–1.01)	0.051
WBC	1.09 (1.05–1.13)	0	1.06 (1.03–1.09)	0
MCV	1.06 (1.03–1.09)	0	1.03 (1.00–1.06)	0.030
RDW	1.18 (1.04–1.32)	0.007	1.12 (1.01–1.23)	0.029
BUN	1.03 (1.01–1.05)	0.012	1.01 (1.00–1.03)	0.060
Calcium	0.74 (0.59–0.93)	0.009	0.72 (0.57–0.91)	0.007
Bicarbonate	1.00 (0.95–1.05)	0.994	0.97 (0.93–1.02)	0.218
Hyperlipidemia	0.62 (0.44–0.88)	0.009	0.65 (0.46–0.92)	0.014

OR, odds ratio; CI, confidence interval; SpO_2_, peripheral oxygen saturation; WBC, white blood cell; MCV, mean corpuscular volume; RDW, red cell distribution width; BUN, blood urea nitrogen.

## 4. Discussion

This is the first study to develop and validate an explicable ML-based prediction model to identify risk factors for 28-day in-hospital mortality of hypertensive ischemic or hemorrhagic stroke patients admitted to the ICU by using data from the eICU-CRD and MIMIC-IV databases. The XGBoost model showed excellent performance (AUC > 0.7) in this study and had good consistency with the LR model in terms of feature importance. Furthermore, the features were segmented into value ranges, making them more suitable for predicting 28-day in-hospital mortality for hypertensive ischemic or hemorrhagic stroke patients admitted to the ICU.

In our study, the 28-day in-hospital mortality was 490 (19%) for hypertensive ischemic or hemorrhagic stroke patients in the eICU-CRD database, which was similar to another cross-sectional study conducted in the USA from 2007 to 2016 (mortality: 21.6%) (14). In our prediction model, the glucose level was the most crucial indicator of in-hospital death, and higher blood glucose levels were associated with increased 28-day in-hospital mortality for hypertensive ischemic or hemorrhagic stroke patients in the ICU. Diabetes is a well-known risk factor for stroke (15, 16). The direct effects of hyperglycemia on brain tissues are possible, but it can also cause microvascular alterations due to an increase in glucose flux, the disruption of intracellular second messenger pathways, an imbalance in the production and scavenging of reactive oxygen species, and advanced glycation of crucial functional and structural proteins (17). In addition to studying the relationship between diabetes and stroke, more and more researchers are paying attention to the relationship between prediabetes and stroke. In the study by Wang et al. (18), prediabetes (plasma glucose concentration between 100 and 125 mg/dL) was significantly associated with the risks of total stroke [hazard ratio (HR) 1.33, 95% confidence interval (CI) 1.18–1.52, *P* = 0.0147] and ischemic stroke [HR 1.33, (95% CI 1.16–1.54), *P* = 0.0413]. In our model, the cutoff value of glucose was 130 mg/dL, which was slightly higher than the normal reference value. Sometimes, clinicians may ignore this slightly elevated blood glucose level. Therefore, our predictive model emphasized this point, which can alert physicians of the severity of the disease for better glycemic management.

Excessive intake of high-cholesterol diet results in elevated blood lipid levels, which causes hyperlipidemia. Numerous studies have shown that hyperlipidemia is a major risk factor for stroke, myocardial infarction, sudden cardiac death, cerebrovascular accidents, and other conditions (19, 20). Studies during the last decade suggest that hyperlipidemia is associated not only with the occurrence of stroke but also with the prognosis of patients after stroke (21–23). This is probably because patients with hyperlipidemia tend to have lower white matter hyperintensity volumes, which have been shown to forecast the progression of infarcts after stroke and result in less favorable clinical outcomes (21, 24). In our prediction model, the risk of death was higher in hypertensive stroke patients with hyperlipidemia, which is consistent with previous studies (23).

According to the model's feature importance, age significantly influenced predictive models. Stroke primarily affects older adults, particularly those over 65, and age significantly affects their prognosis (25). Multiple studies have shown that older adult patients have a higher mortality rate and a lower quality of life following stroke than younger patients (26). In our study, when age was higher than 71. 27 years, it indicated an adverse outcome. In addition, another demographic indicator, ethnicity, also played an important role. Our model, as well as other pieces of evidence (27, 28), suggested that genetic studies could help differentiate stroke subtypes and even assist in patient management.

In this study, several laboratory tests, such as WBC, calcium, MCV, RDW, BUN, and bicarbonate, played important roles in our prediction model. Specifically, WBC is often associated with inflammation. Neuroendocrine hormones that are discharged during an immediate stressful situation can cause an immunological response in stroke patients with an elevated WBC count (29). Zheng et al. (30) found that elevated WBC on admission was associated with death and major disability at 3 months after acute ischemic stroke, and the association was linear (*P* for linear trend = 0.001). However, in another study, the association between WBC count and death at 3 months was not significant (*P* = 0.426) in patients with intracerebral hemorrhage after adjusting confounding factors such as age, sex, and glucose. In our subgroup analysis, an elevated WBC count was associated with increased in-hospital mortality both in ischemic and hemorrhagic stroke. Emerging data about calcium indicate that abnormalities in blood calcium are associated with the risk of stroke (31) and mortality in patients with coronary heart disease (32, 33). In our model, when the serum calcium was <8.8 mg/dL, it indicated an adverse outcome. Furthermore, MCV, RDW, BUN, and bicarbonate substantially contributed to our model. Elevated MCV, RDW, and BUN levels have been associated with increased in-hospital mortality. Additionally, lower bicarbonate, which may suggest metabolic acidosis, indicated a higher risk of mortality.

The characteristics of vital indicators such as SpO_2_ were observed to also affect most predictive models. Specifically, SpO_2_ is a crucial physiological metric for determining how much oxygen is supplied to the human body. It quantifies the proportion of oxygenated hemoglobin to the total hemoglobin. In our prediction model, the cutoff value was 98%, which is generally considered clinically normal. Moreover, in the subgroup analysis, SpO_2_ was not significant in the intracerebral hemorrhage group. Therefore, further research is required in this area in the future.

In this study, to precisely identify 28-day in-hospital mortality for hypertensive ischemic or hemorrhagic stroke patients admitted to the ICU, supervised ML models, such as the ANN, GBM, LR, SVM, and XGBoost, were employed. However, the ML model's operation is in a black box state. In this study, the model with the best average prediction performance on the testing set was considered the best model. This study developed an IML model based on the XGBoost model. Therefore, by developing an interpretable ML model using the shapviz and caret packages, we established the model's ability to depict key features and constructed a high-accuracy mortality prediction model for hypertensive ischemic or hemorrhagic stroke patients admitted to the ICU. The interpretation of feature importance was illustrated by plotting for feature importance and SHAP value. The 11 most important features in descending order were glucose, age, SpO_2_, WBC, ethnicity, calcium, BUN, MCV, RDW, bicarbonate, and hyperlipidemia in the XGBoost model, which mainly resembled the important features of the LR model. Meanwhile, the multi-dimensional correlations between the characteristics of the patients and their outcomes were addressed through regularization and normalization before developing the ML model. These approaches helped generate the ML model that could significantly improve the accuracy of determining mortality risk in hypertensive ischemic or hemorrhagic stroke patients.

This study has several limitations. First, this was a retrospective study using publicly available data; therefore, prospective studies are still needed to further verify our findings. Second, the cutoff values of the most important features were found in this study, but further research is needed to segment the feature values according to the degree of risk. Third, patients with liver disease, renal failure, or respiratory failure were not included in this study; therefore, the prediction model may not be applicable to ischemic and hemorrhagic stroke patients complicated with these conditions.

## 5. Conclusion

This study developed an IML model for predicting 28-day in-hospital mortality in hypertensive ischemic or hemorrhagic stroke patients in the eICU-CRD and MIMIC-IV databases. The 11 most important features in the ICU, including glucose, age, SpO_2_, WBC, ethnicity, calcium, BUN, MCV, RDW, bicarbonate, and hyperlipidemia, were applied in the XGBoost model. The value of these features in predicting the mortality of hypertensive ischemic or hemorrhagic stroke patients was deemed worthy of clinicians' attention. The ML model developed in this study has potential in clinical practice, in that it can help personalize the prevention and strengthen therapeutic strategies.

## Data availability statement

The data analyzed in this study was obtained from the Medical Information Mart for Intensive Care IV (MIMIC-IV) Database, the following licenses/restrictions apply: To access the files, users must be credentialed users, complete the required training (CITI Data or Specimens Only Research), and sign the data use agreement for the project. Requests to access these datasets should be directed to PhysioNet, https://physionet.org/, doi: 10.13026/6mm1-ek67. Publicly available datasets were analyzed in this study. This data can be found at: Electronic Intensive Care Unit (eICU) Collaborative Research database (eICU-CRD), https://eicu-crd.mit.edu/.

## Ethics statement

Ethical review and approval was not required for the study on human participants in accordance with the local legislation and institutional requirements. Written informed consent from the patients/participants or patients/participants' legal guardian/next of kin was not required to participate in this study in accordance with the national legislation and the institutional requirements.

## Author contributions

JH and XL were responsible for conceiving the study. JD, TS, CY, MD, LF, and KW collected the data. HC, SZ, and JH were responsible for writing and revising the manuscript. SZ was responsible for designing the study and APC charge. All authors contributed to the article and approved the submitted version.
