# Supplementary material for: Interpretable machine learning for predicting 28-day all-cause in-hospital mortality for hypertensive ischemic or hemorrhagic stroke patients in the ICU: a multi-center retrospective cohort study with internal and external cross-validation
Source: Front Neurol. 2023 Aug 8;14:1185447. doi: 10.3389/fneur.2023.1185447 (PMC10443100; doi:10.3389/fneur.2023.1185447)
Supplement: Supplementary file 1 [file Table_1.docx]

Supplement Table 1 All baseline characteristics of external validation set

| Variables | Total (n = 1748) | Survival (n = 1473) | Death (n = 301) | p |
| --- | --- | --- | --- | --- |
| ethnicity, n (%) |  |  |  | 0.617 |
| WHITE | 557 (32) | 462 (32) | 83 (30) |  |
| Non-white | 1191 (68) | 985 (68) | 192 (70) |  |
| Bicarbonate,Median (Q1,Q3) | 25 (23, 27) | 25 (23, 27) | 25 (22, 27) | 0.089 |
| BUN, Median (Q1,Q3) | 18 (13, 24) | 18 (13, 24) | 19 (14, 26) | 0.023 |
| Ca, Median (Q1,Q3) | 8.9 (8.5, 9.3) | 8.9 (8.5, 9.3) | 8.9 (8.4, 9.2) | 0.046 |
| glucose, Median (Q1,Q3) | 131 (108, 169) | 127 (106, 162) | 154.5 (124.5, 204.75) | < 0.001 |
| WBC, Median (Q1,Q3) | 9.6 (7.4, 12.4) | 9.3 (7.2, 11.97) | 11.05 (8.3, 14.85) | < 0.001 |
| MCV, Median (Q1,Q3) | 90 (86, 93.5) | 89.85 (86, 93) | 91 (87, 94.95) | < 0.001 |
| RDW, Median (Q1,Q3) | 13.9 (13.2, 14.9) | 13.9 (13.2, 14.8) | 14.1 (13.4, 15.28) | 0.004 |
| spo2, Median (Q1,Q3) | 98 (96, 100) | 98 (96, 100) | 99 (97, 100) | 0.002 |
| hyperlipidemia, n (%) |  |  |  | 0.526 |
| No | 1346 (77) | 1108 (77) | 216 (79) |  |
| Yes | 402 (23) | 339 (23) | 59 (21) |  |
| Stroke, n (%) |  |  |  | 0.002 |
| ischemic stroke | 832 (48) | 665 (46) | 155 (56) |  |
| hemorrhagic stroke | 916 (52) | 782 (54) | 120 (44) |  |
| gender, n (%) |  |  |  | 0.995 |
| female | 841 (48) | 698 (48) | 132 (48) |  |
| male | 907 (52) | 749 (52) | 143 (52) |  |
| Age, Median (Q1,Q3) | 69 (58, 79) | 68 (58, 79) | 72 (60, 81) | 0.005 |
